# Electric field-coupled two-photon polymerization system for on-demand modulation of 3D-printed structural color

**DOI:** 10.1093/pnasnexus/pgaf074

**Published:** 2025-05-13

**Authors:** Wei Feng, Shurong Sheng, Jiaqing He, Xiaopu Wang, Jiaqi Zhu, Jiangfan Yu, Jianhua Zhang, Fan Wang, Li Zhang, Metin Sitti

**Affiliations:** CAS Key Laboratory of Mechanical Behavior and Design of Materials, Department of Modern Mechanics, Institute of Humanoid Robots, University of Science and Technology of China, 96 Jinzhai Road, Hefei, Anhui 230027, China; Hefei Comprehensive National Science Center, Institute of Artificial Intelligence, Hefei 230088, China; CAS Key Laboratory of Mechanical Behavior and Design of Materials, Department of Modern Mechanics, Institute of Humanoid Robots, University of Science and Technology of China, 96 Jinzhai Road, Hefei, Anhui 230027, China; Shenzhen Institute of Artificial Intelligence and Robotics for Society (AIRS), The Chinese University of Hong Kong, Shenzhen, Guangdong 518129, China; Department of Mechanical and Automation Engineering, The Chinese University of Hong Kong, Hong Kong 999077, China; Shenzhen Institute of Artificial Intelligence and Robotics for Society (AIRS), The Chinese University of Hong Kong, Shenzhen, Guangdong 518129, China; Physical Intelligence Department, Max Planck Institute for Intelligent Systems, Stuttgart 70569, Germany; Physical Intelligence Department, Max Planck Institute for Intelligent Systems, Stuttgart 70569, Germany; Department of Mechanical and Automation Engineering, The Chinese University of Hong Kong, Hong Kong 999077, China; Chow Yuk Ho Technology Center for Innovative Medicine, The Chinese University of Hong Kong, Hong Kong 999077, China; Department of Surgery, The Chinese University of Hong Kong, Hong Kong 999077, China; CUHK T Stone Robotics Institute, The Chinese University of Hong Kong, Hong Kong 999077, China; Physical Intelligence Department, Max Planck Institute for Intelligent Systems, Stuttgart 70569, Germany; School of Medicine and College of Engineering, Koç University, Istanbul 34450, Turkey

**Keywords:** two-photon polymerization, 3D printing, structural color

## Abstract

Advanced manufacturing has been extensively studied using various resin monomers and customized apparatus. Multimaterial microfabrication tools remain limited due to the size constraints inherent in extrusion-based fabrication methods. In addition, prior research predominantly employs monomers as “inert” resins, with minimal emphasis on altering their properties during fabrication. In this study, we propose a novel approach to field-coupled advanced manufacturing, wherein external stimulative fields are integrated to dynamically modulate the properties of “dynamic” resins during 3D printing. As a demonstration, we utilize an electric field-coupled two-photon polymerization (EF-TPP) technique to fabricate structurally colorful microstructures. To address the challenges of limited fabrication approach and resins in the field of structural color, we present an EF-TPP system that enables the production of 3D structural colorful microstructures. By coupling the electric field with the two-photon polymerization (TPP) process, this method enhances 3D printing capabilities, allowing for the bottom-up fabrication of structural colorful microstructures. Furthermore, it integrates existing electrically tunable heliconical cholesteric liquid crystals, enabling the modulation of structural color during printing while also accelerating the printing speed. This approach facilitates the production of microstructures with multiple structural colors without requiring changes to the resin ink. By eliminating the lithography step, the EF-TPP system promotes green manufacturing practices and introduces an unconventional paradigm for fabricating dynamic, microscale structural colorful devices. Additionally, the electric field-integrated two-photon lithography system provides a foundational strategy for advancing field-coupled manufacturing methodologies.

**Significance Statement**

Integration of responsive materials into fabrication systems for real-time property adjustment remains underdeveloped. Such integration would enhance printing flexibility and broaden application potential. We introduce the concept of field-coupled advanced manufacturing in the field of structural color, exemplified by an electric field-coupled two-photon polymerization system for fabricating structurally colorful microstructures. Structurally colorful 3D microstructures hold promise in optics and anticounterfeiting applications. Our system leverages an electric field to modulate the self-assembly of liquid crystals during fabrication. We can dynamically tune heliconical cholesteric liquid crystalline (LC) inks with structural color on demand during the 3D printing process. Furthermore, the electric field’s ability to modulate the birefringence of liquid crystals eliminates the need for lithographic steps, offering a versatile and efficient pathway for manufacturing structurally colorful devices.

## Introduction

Advanced manufacturing has garnered significant attention due to the growing practical demand for fabricating complex 3D objects. Through the design of printing paths in extrusion-based 3D printing or light-exposure volumes in stereolithography 3D printing, objects with intricate 3D geometries can be successfully fabricated (1–3). To achieve multifunctionality, the integration of multiple materials into 3D-printed devices is often essential. At the macroscale, multimaterial 3D printing is relatively straightforward. Techniques such as sequential material feeding, customized nozzles in extrusion-based printing, or feed changes in stereolithography and digital light processing enable the incorporation of different materials within a single device (4–8). In contrast, the fabrication of microscale 3D structures with multiple materials presents considerable challenges. Extrusion-based methods are inherently unsuitable for microscale fabrication due to the limitations imposed by nozzle size, which is typically too large for printing microstructures. Similarly, while step-by-step lithography methods such as two-photon polymerization (TPP) are widely used for microfabrication, they pose significant challenges in multimaterial integration. Each lithography step requires precise realignment, a process that is both tedious and technically demanding, despite the high quality of the resulting microstructures (9). Consequently, the limitations of current fabrication instruments remain a major bottleneck in achieving multimaterial microfabrication.

To address the challenges of multimaterial microfabrication, integrating microfabrication tools with other techniques or fields presents a promising strategy. Such integrated systems can be achieved by selectively configuring aspects of the ink, such as magnetic properties, or by employing sequential material feeding (10, 11). For instance, Hu et al. (10) developed a TPP system integrated with magnetic fields to position and align magnetic Janus microparticles, subsequently cross-linking them into a network. Mayer et al. (12) combined a microfluidic system with TPP, enabling multimaterial microprinting where multiple inks could be incorporated into a single microstructure. These innovations, utilizing delicate electromagnetic coils and microfluidics, facilitated the microfabrication of structures with multiple materials.

Despite these advancements, the materials used in most studies were predominantly treated as “inert” during fabrication, ignoring the stimuli-responsive properties. This limitation prevents full utilization of their potential. Stimuli-responsive materials exhibit dynamic properties such as tunable color, mechanical stiffness, and chemical reactivity. These properties can be fixed within the structure during fabrication, enabling the creation of heterogeneous and multifunctional devices. Consequently, field-coupled additive manufacturing offers significant advantages by incorporating responsive materials as resins. One notable example of responsive materials in field-coupled TPP is the fabrication of liquid crystalline (LC) microstructures with heterogeneous director distributions (13, 14). By applying an electric field during TPP, arbitrary spatial programming of LC directors within a monolithic 3D microstructure was achieved, demonstrating potential applications in robotics. However, the application of field-coupled TPP remains underexplored in the context of other fields, e.g. structurally colorful microstructures, as summarized in Table S1. The applications of structurally colorful microstructures are particularly hindered by low printing efficiency.

Structurally colorful microstructures have a wide range of applications, driving significant research interest in their fabrication methods. TPP systems, which employ femtosecond lasers, provide high spatial resolution and enable spatially controlled chemical reactions, such as chemical bonding (15, 16) and polymerization (17, 18), for the fabrication of intricate microstructures. TPP has thus become a widely adopted technique for producing microscale structural colorful structures. Fabrication strategies for 3D microscale structural colorful structures generally fall into two categories: top-down and bottom-up approaches. The top-down strategy involves directly mimicking natural structures by printing woodpile lattice-type architectures or nanopillar gratings, typically with periodicities in the visible-light range (400–800 nm) (19–21). While effective, this method requires specialized high-resolution resins and involves extended printing times, which can limit its practicality. The bottom-up strategy, on the contrary, leverages self-assembling materials that spontaneously form periodic nanostructures, such as colloidal crystals or liquid crystals (22–25). Although promising, challenges yet remain for these methods. Colloidal crystals often require prefixation to prevent disruption from laser exposure, while liquid crystal-based approaches are constrained by the predetermined concentrations of chiral dopants. As a result, the ability to tune and fix structural colors in real time during printing remains highly desirable for advancing these methods. Additionally, both strategies typically require postprint lithographic processing to remove uncured resins. This process is essential because the structural colors arise from differences in refractive indices between the printed resin and air. However, lithographic developers such as 1-methoxy-2-propanol acetate (PGMEA) are hazardous and pose significant health risks to operators with prolonged use. For instance, glycol ethers pose considerable health risks, including male and female reproductive hazards and the potential to cause pancytopenia (26, 27). Additionally, methanol, another common solvent, is associated with visual field disturbances, further underscoring the need to eliminate hazardous postprocessing steps in microfabrication techniques. The primary bottleneck in achieving this lies in the lack of suitable resins that can enable direct printing of structural colorful microstructures without requiring lithography.

In this article, we introduce an electric field-coupled two-photon polymerization (EF-TPP) approach for advanced manufacturing, demonstrating its application in the microfabrication of structurally colorful microstructures. The electrically responsive resin utilized in this approach consists of oblique helicoidal cholesteric (Ch_OH_), also referred to as heliconical cholesteric liquid crystals (28). Following the printing of structurally colorful microstructures, the remaining resin can be switched to a nonbirefringent homeotropic state to serve as a colorless structural background. This transition is achieved by applying a sufficiently strong electric field to switch the heliconical liquid crystal into the homeotropic state (29). A flood UV-initiated polymerization process can solidify the nonbirefringent homeotropic LC resin, obviating the need for additional lithographic steps. The novelty of our approach is 2-fold. (i) By integrating an electric field with TPP, we achieve real-time tuning of the structural color of the resin during the printing process. This significantly enhances printing efficiency compared to grating-based top-down strategies. (ii) Our method is more environmentally friendly and safer for operators, as it eliminates the need for a lithographic step. This approach represents the first demonstration of in situ structural color modification during 3D printing, thereby advancing the methodology of additive manufacturing and expanding its potential applications.

## Results

### Design of the EF-TPP system

TPP typically employs compositionally static resins, including commercial resins such as IP-L and IP-S, as well as SU-8, acrylamide, or pentaerythritol tetraacrylate (30–32). However, few studies have explored the modification of resin properties during the polymerization process. This limitation arises primarily because most stimulation processes for responsive resins involve either the addition of chemicals (e.g. for pH- or redox-responsive materials) or the application of light (e.g. for photoresponsive materials) (33–35). Introducing stimulative chemicals is often impractical in conventional TPP workflows, while external light fields, however, can interfere with the TPP laser and prematurely initiate polymerization. Given these challenges, integrating remotely controllable electric or magnetic fields to modulate resin properties during TPP is a more feasible and desirable strategy. The oblique helicoidal cholesteric state of the liquid crystals used in this study has been experimentally confirmed in previous work by Lavrentovich et al. (36). The key contribution of the present work lies in the development of an EF-TPP system, which enhances the conventional TPP apparatus by introducing greater tunability and versatility for fabricating structurally colorful microstructures. In our EF-TPP system, the strength of the applied electric field can be adjusted to modulate the periodic pitch of the polymerizable resin, thereby altering the structural color observed under white light. The resin used in this system consists of a polymerizable acrylate formulation, which is well suited for the TPP process. This methodological advancement simplifies the fabrication of structurally colorful microstructures and provides a robust framework for integrating responsive materials into TPP-based manufacturing.

**Fig. 1.**
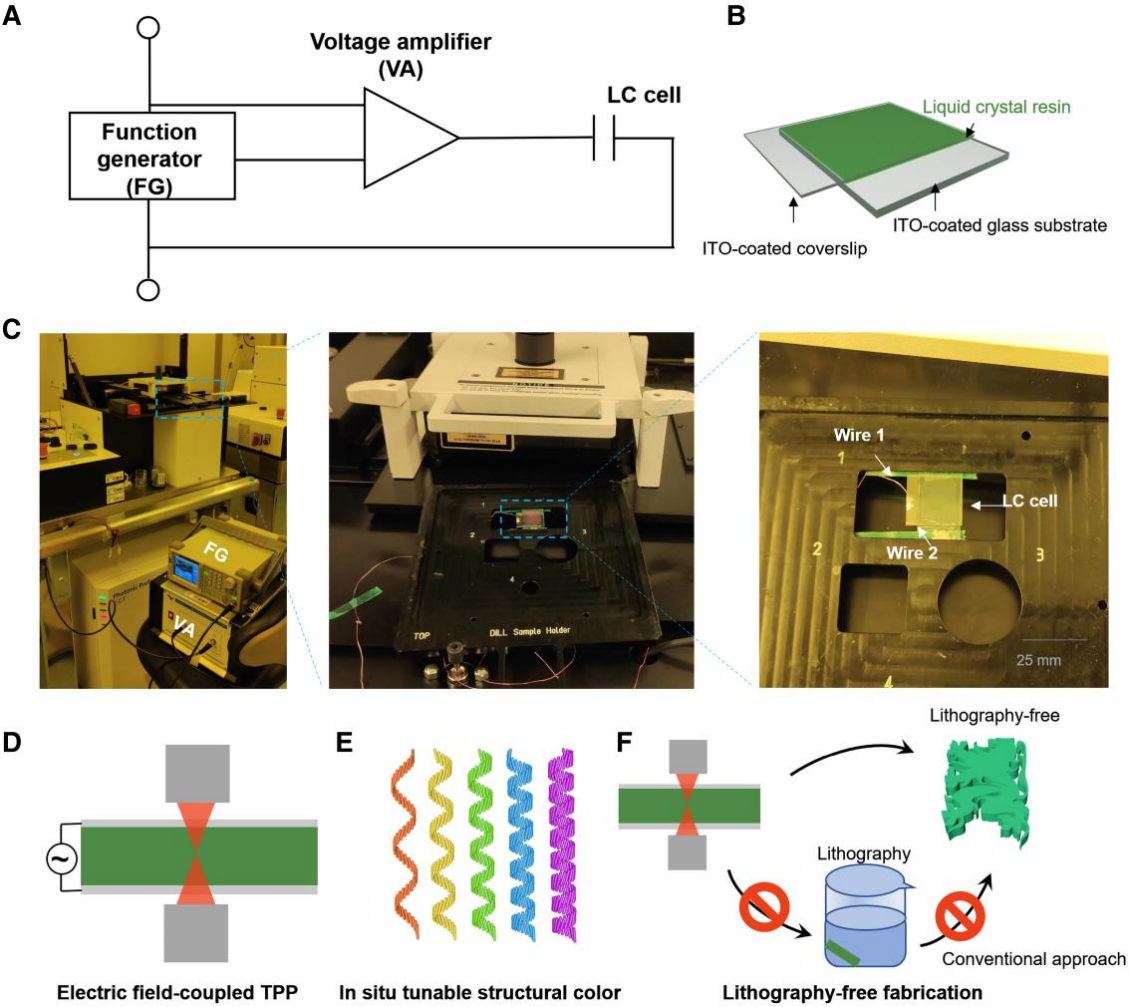
Design of the EF-TPP system. A) Circuit diagram of the EF-TPP system. The electrical signal from the function generator is amplified via a voltage amplifier and input to the LC cell loaded in the TPP system. The LC cell is abbreviated as a capacitor as it is composed of two ITO-coated glasses with LC resin sandwiched. B) Schematic illustration of the LC cell. The LC resin is sandwiched between ITO-coated coverslip and glass substrate. C) Images of the EF-TPP system. The liquid crystal cell is loaded in the sample loader and connected to external functional generator and voltage amplifier. Wires are soldered on the ITO side of the coverslip and glass substrate. D) Schematic illustration of the printing process with TPP system. E) The structural color of monomeric resins is tuned electrically while printing. The strength of applied electric field is altered to tune structural color of the heliconical cholesteric LC resin. F) With the EF-TPP system, the printing of structural colorful microstructures can be processed in a lithography-free manner without using developers.

Here, we develop the EF-TPP system by integrating a commercial TPP setup (Photonic Professional GT2, Nanoscribe GmbH) with a function generator (RSDG805, RS Components Ltd) and a voltage amplifier (WMA-300, Falco Systems), as illustrated in Fig. 1A. A square wave electric signal with a frequency of 1 kHz is generated by the function generator, subsequently amplified by the voltage amplifier, and applied to the LC cell (Fig. 1B and C). Within the LC cell, a thin indium tin oxide (ITO)-coated coverslip (∼170 µm thick) is employed to confine the LC resin to the working distance of 380 µm from the TPP focal point.

In our EF-TPP system, the choice of electrically responsive ink is also a critical consideration. Helicoidal cholesteric liquid crystals previously were used for fabricating structurally colorful devices (5, 37, 38). However, these studies primarily relied on techniques such as ink direct writing, film casting, or capillary filling, limiting the resulting devices to millimeter-scale dimensions. Although a few reports have utilized helicoidal cholesteric liquid crystals as resins for TPP (24), the development of compositionally dynamic resins tailored for TPP remains unexplored.

In this study, we select oblique helicoidal liquid crystals composed of the bent-shaped liquid crystal CB7CB as the electrically responsive resin for the EF-TPP system (Fig. 1D and E). This resin exhibits electrotunable structural color by changing helical pitch. The underlying mechanism is that CB7CB features a bent molecular conformation and has a bend elastic constant *K*_3_ smaller than the twist elastic constant *K*_2_ (*K*_3_ < *K*_2_) (28). This unique elastic property drives the liquid crystal molecules to align under external fields with a tilt angle smaller than π/2 relative to the helicoidal axis, making it possible to tune via electric field (29, 36, 39). In the absence of electric fields, heliconical cholesteric liquid crystals exhibit a “focal conic” texture, as shown in Fig. S1. The structural color of these liquid crystals can be dynamically adjusted by modulating the electric field strength applied in the customized TPP system, transitioning from violet to green to red as the field strength decreases. After microfabrication, the LC resin can be aligned into a homeotropic orientation, eliminating the need for conventional lithographic processes (Fig. 1F).

Additionally, the presence of birefringent resins would modify the optical writing field. Previously, Münchinger et al. (13) used optical phase plate and half-wave plate to ensure the single well-defined laser focus for the uniaxially aligned LC resin. In our case, the resin is heliconical cholesteric LC resin where the LC mesogens form oblique helical structures. The laser employed in the process operates at a wavelength of 780 nm, which lies outside the reflection band of the heliconical cholesteric liquid crystal (spanning the visible-light range of 400–700 nm). The linearly polarized light emitted by the two-photon polymerization instrument would undergo modulation upon interaction with the heliconical cholesteric LC resin, leading to substantial rotation of the major axis of polarization and a transformation into circularly polarized light within the resin (40) following the equation:


$$\rho =-\frac{2\pi \alpha^{2}}{8P_{0}{\left(\lambda_{0}/\lambda_{B}\right)}^{2}[1-{\left(\lambda_{0}/\lambda_{B}\right)}^{2}]}$$

where $\alpha^{2}=(((n_{∥}^{2}-n_{⊥}^{2}{)}^{2})/(n_{∥}^{2}+n_{⊥}^{2}))$, *ρ* is the twisting power of chiral dopant, $\lambda_{0}$ is the wavelength of laser beam, *P_0_* is the pitch of the helix, and $\lambda_{B}$ is the central wavelength of reflection band. Although the heliconical cholesteric LC resin alters the writing optical field, this alteration does not cause multiple foci and would not substantially affect the printing outcome. Consequently, optical corrections are not implemented in our experiments.

### Printing parameter investigation

**Fig. 2.**
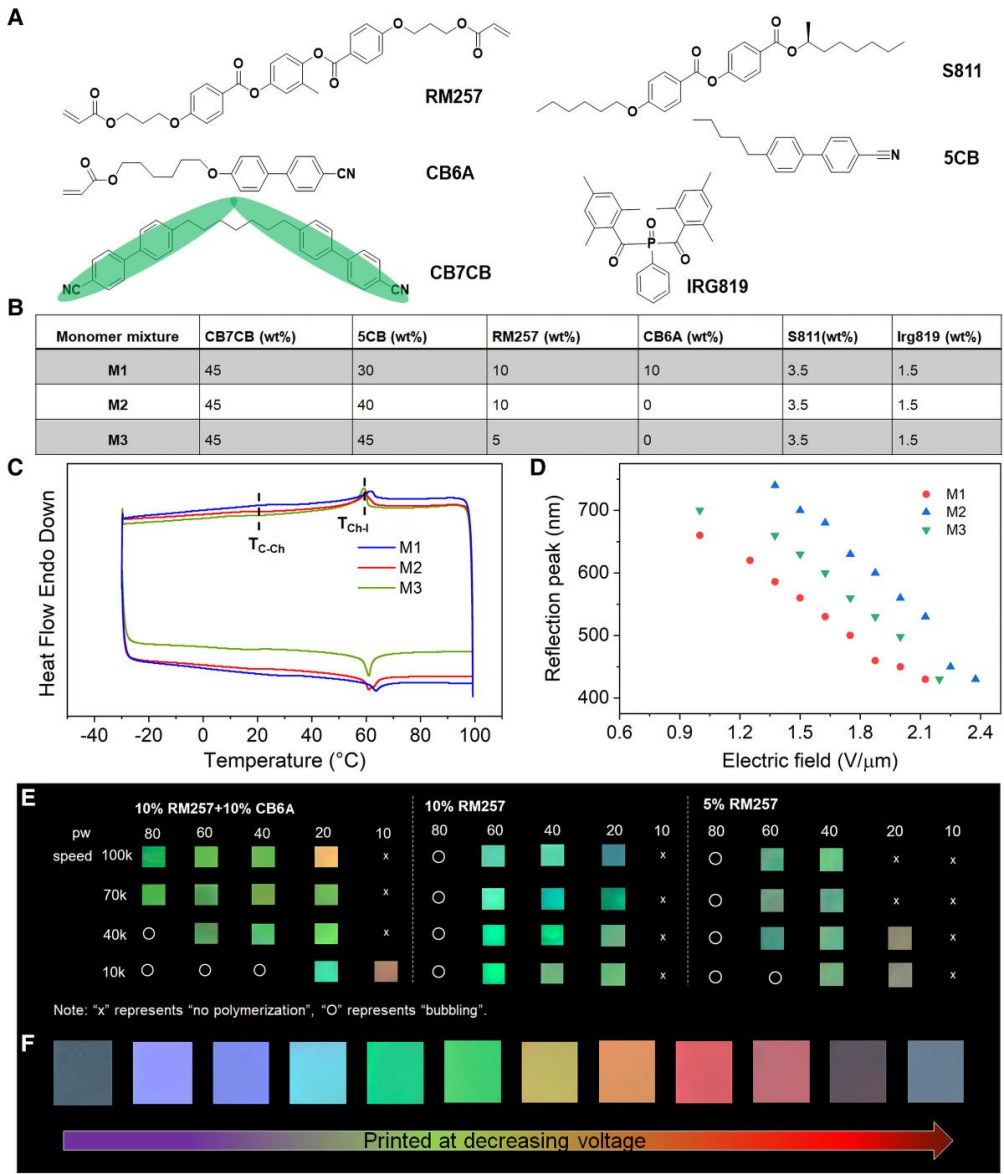
Investigation of material and printing parameters of EF-TPP using heliconical cholesteric liquid crystals. A) Chemical structures of LC mesogens. RM257 and CB6A are polymerizable mesogens, while CB7CB manifests the LC resin as a heliconical cholesteric phase. Chiral compound S811 endows the mixture with uniform handiness. 5CB widens the LC phase of the monomer to room temperature. B) Formulated monomer sets with different amounts of LC components. The amounts of polymerizable RM257 and CB6A are varied to achieve optimal printing effect. C) DSC curves of monomer mixtures reveal LC phase at room temperature. D) Reflection data of different formulations at varied driving electric fields. All three resin sets can exhibit structural color in the visible-light range upon electrical stimulation. E) Influence of printing parameters on the structural color of microstructures formed by different resin formulations. The driving electric field is fixed for these tests. The used laser power is set in the form of a percentile of the full power (50 mW). Unit of scan speed: µm/s. F) Various colors can be printed in microstructures by varying the driving electric field.

We formulate polymerizable LC mesogens RM257 and CB6A into a photoresist system (Fig. 2A). The CB7CB content is fixed at 45 wt% (Fig. 2B) to ensure the formation of the oblique cholesteric phase and to regulate the structural color and phase transition temperature of Ch_OH_ (39, 41, 42). Chiral dopant S811 is incorporated to induce uniform helicity, while mesogen 5CB contributes to widening the LC temperature range. The polymerizable reactive mesogen RM257 functions as a cross-linker, and CB6A, with its large dipole moment, enhances the system's responsiveness. The dipole moments of these LC mesogens are calculated using density functional theory (DFT) simulations (Table S2). Similar formulations were reported previously but for fabricating 2D structures (43), and the fabrication of 3D microstructures remains unexplored. The novelty of this study lies in the integration of electric fields and TPP for the advanced manufacturing of 3D microstructures.

The printing parameters for heliconical LC resins are systematically evaluated across different formulations. Variations in the concentrations of RM257 and CB6A are employed to modify the resins’ cross-linking densities. Differential scanning calorimetry (DSC) measurements confirm that all three resin formulations exhibit a LC phase at 25 °C, with a crystal-to-cholesteric phase transition temperature *T*_C–Ch_ of 20 °C and a cholesteric-to-isotropic phase transition temperature *T*_Ch–I_ of 61 °C (Fig. 2C). These inks also demonstrate electrically tunable structural colors (Fig. 2D). The underlying mechanism is that the helical pitch of ChOH liquid crystal microstructures can be modulated by adjusting the electric field strength. This tunability arises from their mechanical properties, specifically the bend elastic constant *K*_3_ being lower than the twist elastic constant *K*_2_ (*K*_3_ < *K*_2_) (28) as discussed earlier. In contrast, conventional helicoidal liquid crystals do not exhibit this behavior, as they undergo twisting of the helical axis rather than a change in helical pitch. The electric field strength required for structural color tuning varies slightly among the resin sets and is influenced by factors such as the elastic constants of bend *K*_3_ and twist *K*_2_, dielectric anisotropy, and the wavelength of structural color (36, 41, 44). Printing parameters, including scanning speed and laser power, are optimized to achieve the desired printing effects (Fig. 2E). Insufficient laser power results in incomplete printing and altered structural colors, while excessive laser power causes bubbling during fabrication. Among the three monomer sets, resins with higher monomer content display lower power thresholds and broader acceptable power ranges. However, increasing the 5CB content makes the resins more prone to bubbling due to the lower temperature range of the LC phase. Experiments confirm that the entire visible spectrum could be achieved by fine-tuning the driving electric field strength during printing (Fig. 2F). Within the available printing range, the structural color remains consistent owing to the absence of a lithographic step. Unlike conventional lithography, which removes low-molecular-weight oligomers from the microstructures, this lithography-free approach preserves material integrity, ensuring color stability.

**Fig. 3.**
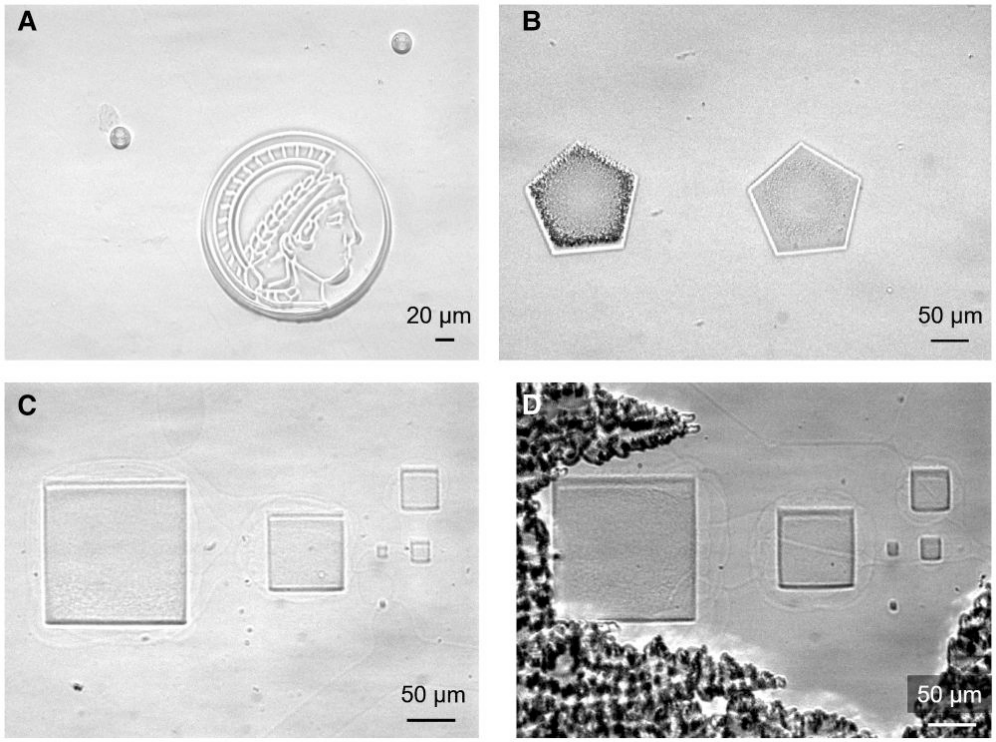
Printing parameter investigation and optimization. A) Microsphere spacers are sputtered in the LC cell assisted by N_2_ flow for uniform spacing and to prevent the deformation of thin glass coverslip. B) Phase separation during printing at different thicknesses. Microstructures of various thicknesses are printed to verify the phase separation process, and phase separation is observed for thick microstructures at high laser power (50 mW). C) No phase separation is observed for optimized printing parameters (laser power: 40 mW, scanning speed: >10,000 µm/s). The texture is relatively uniform for Ch_OH_ phase with sufficiently large driving electric fields. D) Focal conic texture emerges when the electric field is not large enough to drive the assembly of Ch_OH_ liquid crystals.

Notably, the printing parameters are optimized to enhance the quality and precision of the fabricated microstructures. As mentioned earlier, we sputter microparticle spacers in the LC cell to maintain uniform spacing between the relatively flexible glass coverslip and rigid 1-mm ITO-coated glass substrate (Fig. 3A). One potential concern is the influence of light polarization or optical torque exerted by the laser of the TPP system on the heliconical LC structure (44–46). Experimental observations reveal intriguing phenomena associated with this interaction. At high laser power (50 mW), localized heating induced by the TPP laser leads to bubbling in the resin (Fig. S2). Reducing the laser power to 45 mW mitigates bubbling but causes a mass transport phenomenon along the laser scanning direction, likely due to optical torque. This mass transport effect is eliminated by using lower laser power (40 mW).

Another challenge observed is phase separation in the resin system during TPP, which arises from the presence of nonpolymerizable LC mesogens (47–49). Phase separation is particularly pronounced when printing thicker microstructures (>10 µm) at low scanning speeds (∼5,000 µm/s, Fig. 3B), as captured in Movie S1. As observed in the recorded video, the printed microstructure initially exhibits a smooth surface during the early stages of the printing process. However, as printing progresses, real-time imaging using the camera in the TPP system reveals increasing surface roughness, indicating the onset of phase separation. To address this issue, scanning speeds are increased to over 10,000 µm/s, which effectively eliminates visible phase separation (Fig. S3). This behavior can be attributed to the relationship between phase separation and light intensity during TPP. TPP polymerization rate is strongly connected to the light intensity of the scanning laser. The absorption rate of energy is proportional to the square of light intensity following the equation (50, 51):


dWdt=8π2ωn2c2I2Im(χ(3)),

where *I* represents the light intensity, Im(χ(3)) is the imaginary part of the third-order susceptibility, *c* is the speed of light in vacuum, *n* is the refractive index, and ω is the incident light optical frequency. The phase separation is severe at high light intensity and polymerization rate as a faster polymerization rate (increased by higher light intensity) can reduce the time available for components to mix and enhance phase separation (48). Further models may couple the Flory–Huggins theory for phase separation with kinetic equations for polymerization. The specific relationship depends on multiple parameters, including viscosity, diffusion coefficients, and reactivity ratios. The detailed explanation is still under investigation and beyond the main scope of this paper. These optimizations underscore the critical role of fine-tuning laser power and scanning speed to mitigate thermal effects, optical torque, and phase separation, ensuring high-quality 3D microstructure fabrication. The integration of a camera into the TPP system further enhances the process by enabling real-time visualization of phase separation, allowing for immediate adjustments to the printing parameters to avoid such issues. Additionally, the camera facilitates straightforward monitoring of the LC texture and any phase separation during the printing process (Fig. 3C). The observed texture is relatively uniform when the applied electric field is sufficiently strong to induce the oblique cholesteric state. In contrast, a noticeable focal conic texture appears under weaker electric fields (Fig. 3D). Importantly, by comparing the structural color of the monomeric resin with that of the printed microstructures under the same driving electric field, the structural colors remain consistent before and after polymerization. This consistency highlights the reliability and precision of the EF-TPP system in maintaining the intended optical properties of the fabricated microstructures.

In addition to the electrooptical effect, the potential influence of an applied electric field on polymerization, specifically in terms of macromolecular molecular weight and polydispersity, warrants consideration. Previous studies demonstrated that electric fields led to increased molecular weight and narrower molecular weight distributions in the free radical polymerization of isobornyl acrylate and the ring-opening polymerization of epoxides (52, 53). However, the electric field strengths required for these effects (14 V/µm) are significantly higher than those employed in our study (<2.5 V/µm). Furthermore, prior investigations into the polymerization of LC polymers under applied electric fields reported no discernible impact on the actuation performance compared with liquid crystal polymers synthesized without an electric field (13, 54–56).

### Lithography-free manufacturing of 2D and 3D microstructures

**Fig. 4.**
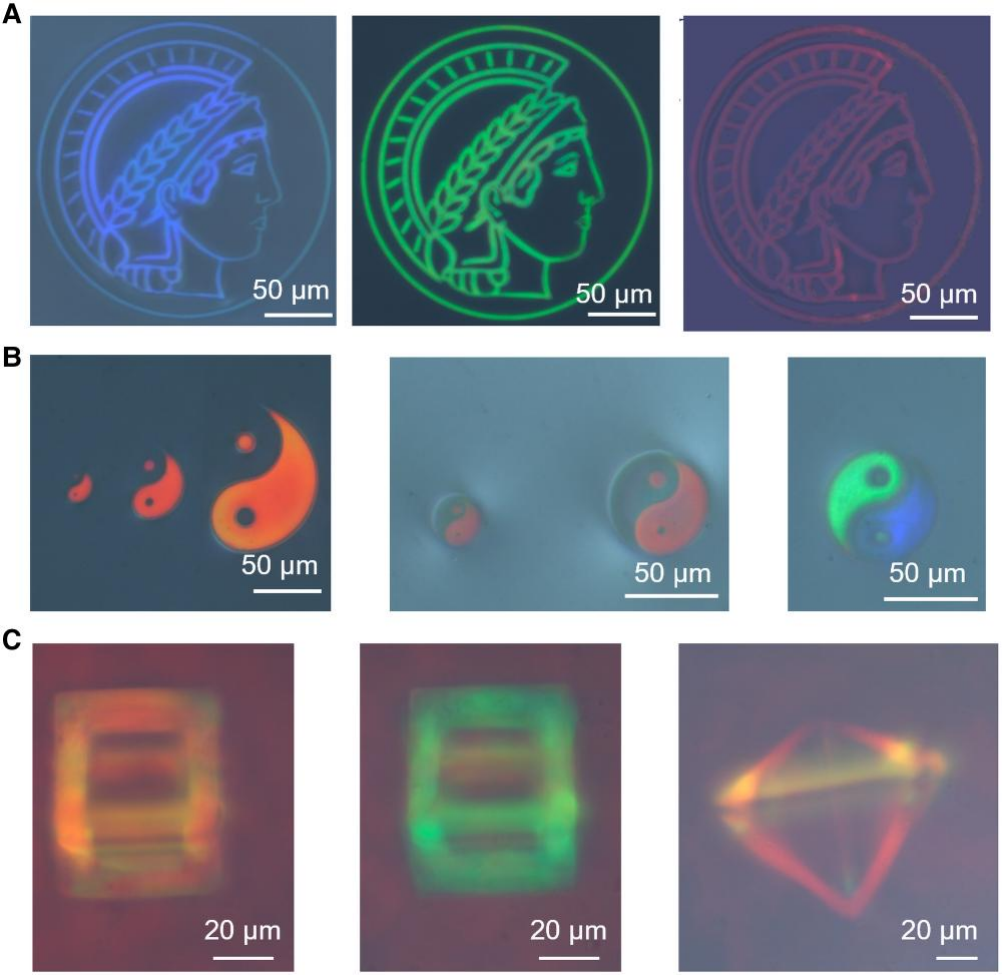
Printed 2D and 3D microstructures in different structural colors. A) Logo of 2D Max Planck Society in different colors. The patterns are printed at different driving electric fields, yielding different structural colors. B) 2D Taiji logo in various colors and combinations. The patterns of different sizes are printed to test the resin's printing resolution limit. A pattern can be printed in different colors by selectively printing certain parts at one driving electric field strength and other parts at a different driving electric field strength. C) 3D microscale frames. The frames are printed in LC cells with a 65-μm gap at different driving electric field strengths.

Conventional lithography methods typically follow a “loading resin-print-develop” protocol, which necessitates the use of chemical developers. These developers, such as glycol ethers or methanol-based solutions, pose significant health hazards to operators through inhalation exposure. Prolonged exposure to such chemicals may lead to adverse effects, including respiratory issues, reproductive hazards, and even pancytopenia (26, 27). This concern underscores the importance of exploring alternative fabrication techniques that minimize or eliminate the need for postprocessing with harmful chemicals. Using the EF-TPP system, we demonstrate the lithography-free fabrication of structural colorful microstructures with tunable helical pitch and structural color. By varying the strength of the applied electric field during printing, we successfully alter the structural color of the resin, enabling the same image to be fabricated in different colors, as illustrated with the MPG symbol in Fig. 4A. Sequential printing with controlled voltage changes allows for distinct colors to be fixed within the same microstructure. For instance, the Taiji symbol is printed in dual colors, with each half interconnected (Fig. 4B). This method also achieves high-resolution printing, as shown by the 4-µm-scale microstructures within the Taiji logo (Figs. 4B and S4). After printing, the uncured heliconical liquid crystal is unwound under a sufficiently strong electric field and polymerized in homeotropic alignment. Importantly, the stable structural colors of the microstructures are preserved throughout UV-initiated polymerization, while the colorless background resin in the homeotropic alignment was polymerized to remain colorless (Fig. S5). These microstructures are kept between glass substrates after preparation, ensuring their shape/dimension stability. Additionally, this approach extends to the fabrication of versatile 3D microstructures, such as 3D microframes (Fig. 4C).

The EF-TPP system enables efficient fabrication of structural colorful microstructures, overcoming the limitations of prior strategies. For example, high-resolution woodpile-type microstructures with lattice periods matching visible-light wavelengths, commonly found in biological tissues, have been mimicked in artificial microstructures (20, 21, 57). However, this approach is highly time-intensive due to the stringent resolution requirements. Fabricating a 50 × 50 × 50 µm^3^ cubic microstructure using the woodpile method takes ∼406.5 min. In contrast, the EF-TPP system drastically reduces this time to just 1.8 min—only 0.44% of the time required by the woodpile strategy (Fig. S6). Furthermore, the switchable structural color demonstrated here during printing is not achievable solely by modifying printing parameters when using helicoidal cholesteric liquid crystals (Figs. S7 and S8).

### Responsive structural colorful microstructures

**Fig. 5.**
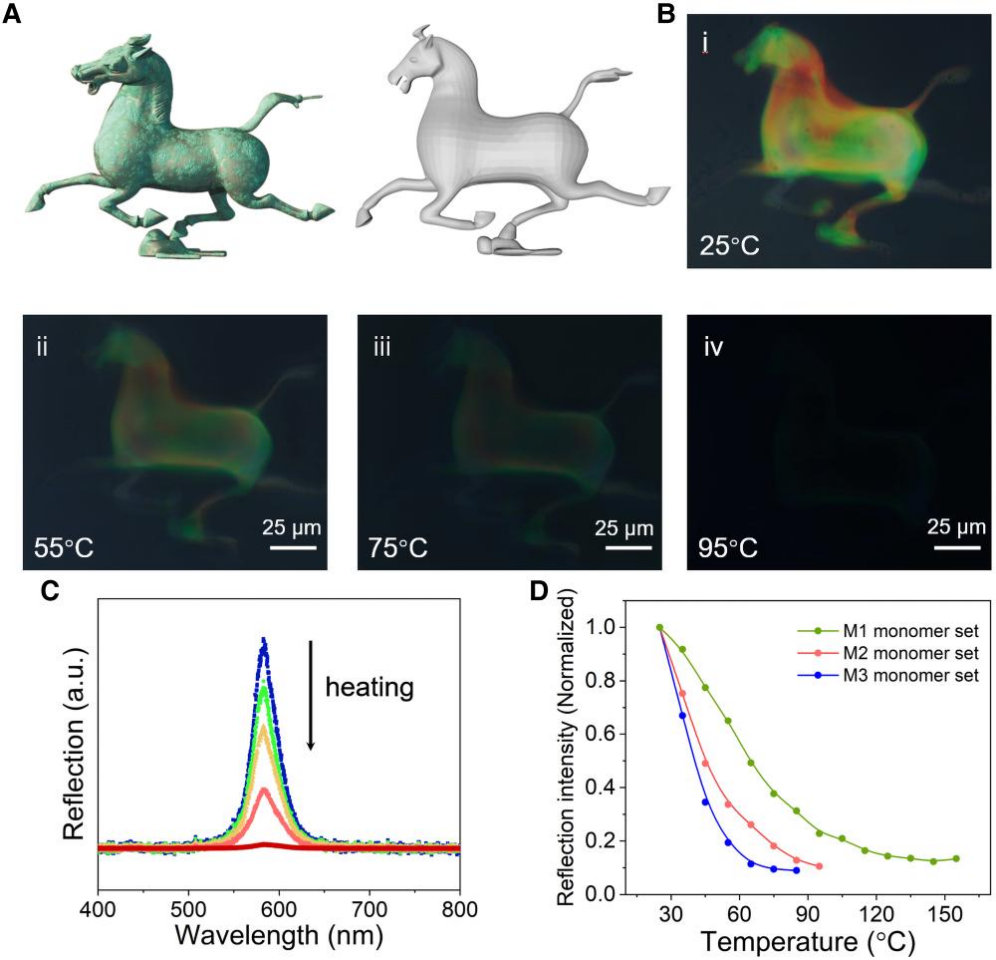
3D microstructure with multiple colors and the thermal response. A) Depiction of the traditional Chinese statue “Galloping Horse Treading on a Flying Swallow” and corresponding 3D model. B) Printed 3D microstructure in combinational colors and its response to thermal stimulation from 25°C to 95°C. C) Changing reflection spectra of the selected structural color with a reflection band at 580 nm during heating of microstructures made from M1 monomer set. D) Quantitative analysis of the structural color of printed microstructures in response to thermal stimulation. Thermal response behaviors of three resins are shown. The microstructure with the fewest cross-linkers exhibits the most thermosensitive behavior.

The tunable intensity of structural colors significantly broadens the application potential of colorful microstructures, including areas such as encryption and light manipulation. Previous studies have shown that the structural color of the Ch_OH_ state in liquid crystals is dependent on temperature or electric field strength (43, 58). Here, we demonstrate that the structural color of the polymerized microstructures fabricated with the EF-TPP system is also responsive to thermal stimuli. As a showcase, we fabricate a microscale replica of the Chinese traditional statue, *Galloping Horse Treading on a Flying Swallow* (Fig. 5A), in multiple colors by selectively printing different regions under varying electric field strengths. The resulting microstructures display a clear response to temperature changes (Fig. 5B). When subjected to heating, the intensity of reflected structural colors gradually diminishes as the temperature increases (Fig. 5C). This phenomenon is attributed to the phase transition of the liquid crystals, transitioning from the ordered LC cholesteric phase to the colorless isotropic phase.

Moreover, the temperature responsiveness of the structural color can be tuned by adjusting the cross-linker content in the resin formulation. Three resin formulations with varying amounts of polymerizable mesogens exhibit different degrees of thermal sensitivity (Fig. 5D). Microstructures with lower polymerized mesogen content show greater thermal responsiveness: microstructures fabricated with the M1 resin recipe demonstrate the highest thermal stability, with their colors fading entirely at 120 °C; in contrast, those made from the M3 recipe exhibit the most pronounced thermal sensitivity, losing their structural color at 60 °C.

Currently, the application of reported heliconical LCs is limited by issues such as crystallization and the narrow temperature range of the twist-bend nematic phase (59, 60). We noticed that polymer-stabilized samples of these LCs begin to crystallize within 2 days at room temperature (25 °C). One possible approach to alleviate this crystallization is to store the printed devices at higher temperatures. Notably, the TPP-printed microstructures exhibit a slower crystallization process compared with the background resin that was polymerized via UV LED flood exposure (Fig. S9). This difference may stem from the variation in polymerization kinetics between TPP and conventional single-photon polymerization, suggesting a potential path for reducing crystallization in future resin formulations. Further efforts can be made in replacing the 5CB with polymerizable mesogens (e.g. RM257 or CB6A), and an extra hot stage can be integrated in the TPP system to address challenges associated with elevated temperature range of LC phase.

### Limitation of the EF-TPP system and potential applications

While the EF-TPP system demonstrates significant advancements in microstructure fabrication, there is still a need to develop more stable resins. Furthermore, the development of stable heliconical LC mixtures, such as those developed by Zheng et al. (61), holds promise for enhancing the temporal stability of polymer-stabilized heliconical LC systems. In addition, several potential applications of the EF-TPP system can be highlighted. The structural colorful microstructures created with this system have significant potential in light manipulation, such as controlling the orbital angular momentum of structured light through microprinted spiral phase plates (62). Moreover, the versatility of the EF-TPP system extends to other materials, including electrically responsive anisotropic hybrid hydrogels and redox-responsive materials (63, 64), opening up opportunities for a wide range of applications beyond structural color.

## Conclusion

We have developed an EF-TPP system capable of efficiently fabricating structural colorful microstructures in a lithography-free manner. By incorporating oblique cholesteric liquid crystals, the fabrication efficiency of these microstructures is significantly enhanced. This approach introduces a novel method for the development of structural colorful microstructures and advances the field of additive manufacturing. Moreover, the EF-TPP system opens new possibilities for other electrically or redox-responsive materials, expanding the capabilities of microfabrication techniques.

## Materials and methods

### Materials

4′,4′′′-(Heptane-1,7-diyl)bis(([1,1′-biphenyl]-4-carbonitrile)) (CB7CB, 98%, Synthon Chemicals), 2-methyl-1,4-phenylene bis(4-(3-(acryloyloxy)propoxy)benzoate) (RM257, 97%, Synthon Chemicals), 6-((4′-cyano-[1,1′-biphenyl]-4-yl)oxy)hexyl acrylate (CB6A, 97%, Synthon Chemicals), (*S*)-2-octyl 4-[4-(hexyloxy)benzoyloxy]benzoate (chiral dopant S811, 98%, TCI), 4-cyano-4′-pentylbiphenyl (5CB, 98%, TCI), and phenylbis(2,4,6-trimethylbenzoyl)phosphine oxide (IRG819, 97%, Sigma-Aldrich) were used as received. ITO-coated coverslips (0.17 mm thick, 18 × 18 mm) were obtained from SPI Supplies. Regular ITO-coated glass substrates (∼1 mm thick) were purchased from Sigma-Aldrich.

#### Substrate preparation and LC cell assembly

ITO-coated coverslips and glass substrates were cleaned by ultrasonication in acetone and n-isopropanol for 5 min, followed by treatment with UV ozone for 20 min. After cleaning, a layer of polyimide (PI2555, Nissan Chemical Corporation) was spin-coated onto the substrates at 5,000 rpm for 1 min. The polyimide layer was then thermally baked first at 100 °C for 10 min, followed by curing at 180 °C for 90 min. The polyimide-coated substrates were then gently rubbed with a cloth to enhance alignment. To set the gap of LC cell, the dry microparticles were gently sputtered from a tiny spoon to the ITO glass substrate assisted by a nitrogen flow (0.1 MPa). The glass substrate was then assembled into a LC cell with an ITO-coated coverslip using adhesive glue (NOA65, Thorlabs) mixed with sphere spacers (microParticles GmbH). The gap size of the LC cell was set to 20 µm using corresponding spacers, unless otherwise specified; for 3D frame fabrication, the gap was set to 65 µm.

#### LC monomer preparation

LC monomers were weighed according to a designated ratio, mixed in dichloromethane, and dried in a vacuum at 45 °C to remove the solvent. The LC monomer ink was then heated to 80 °C to reach the isotropic phase before being filled into the LC cell via capillary action.

#### EF-TPP system setup

The EF-TPP system was realized by integrating a commercial TPP system (Photonic Professional GT2, Nanoscribe GmbH) with an electrical setup. A 1-kHz square wave electric signal generated by a function generator (RSDG805, RS Components Ltd.) was amplified using a voltage amplifier (WMA-300, Falco Systems) and applied to the LC cell, which consisted of an ITO-coated coverslip (∼170 µm thick) and an ITO-coated glass substrate (∼1 mm thick). Copper wires (0.1 mm diameter) were soldered to the ITO-coated substrates using indium paste as the bonding material.

#### Sample orientation and microfabrication setup

The LC cell was oriented such that the coverslip side faced the objective lens of the TPP system. One of the substrates was a thin ITO-coated coverslip (∼170 µm thick), ensuring that the LC resin remained within the working range of the TPP system. The sample was processed under oil immersion configuration using a 25× magnification objective (Zeiss LCI Plan-Neofluar) with a numerical aperture of 0.8. For the fabrication of 2D patterns, the pattern thickness was set to 10 μm, with slicing and hatching distances both set to 0.2 µm.

#### Printing parameter investigation and optimization

The influence of printing parameters on the resulting structural colors was examined by printing identical patterns while varying parameters such as laser power, scanning speed, and driving electric field strength. Although the glass coverslip exhibited some flexibility, the densely sputtered microparticle spacers ensured uniform gap formation within the liquid crystal cell. To monitor phase separation, the Zeiss AXIO camera integrated into the TPP system captured in situ images using Axio Vision software. Consequently, phase separation was minimized to a negligible extent by optimizing the printing parameters.

### Characterization

#### Structural color measurement

The structural color of the liquid crystal samples was analyzed using a polarized optical microscope (Zeiss AXIO), equipped with crossed polarizers in the reflection mode.

#### Phase transition temperature determination

The liquid crystalline phase transition temperatures were determined by DSC. DSC measurements were taken using a DSC 2500 instrument from TA Instruments, with a temperature ramp rate of 10 °C/min. The obtained DSC curves provided information about phase transitions of the liquid crystal materials, including the transition from the crystal to cholesteric phase and cholesteric to isotropic phase.

#### Optical reflection spectroscopy

The optical reflection spectra of the samples were recorded using a Thorlabs CCS200/M compact spectrometer. This setup allowed for the precise measurement of the reflected light, providing data on the structural color and its dependence on temperature.

## Supplementary Material

pgaf074_Supplementary_Data

## Data Availability

All the data in the manuscript are complete, comprehensive, and original. All data and materials are available in the manuscript and Supplementary Information.
