# Supplementary material for: Electric field-coupled two-photon polymerization system for on-demand modulation of 3D-printed structural color
Source: PNAS Nexus. 2025 May 13;4(5):pgaf074. doi: 10.1093/pnasnexus/pgaf074 (PMC12070393; doi:10.1093/pnasnexus/pgaf074)
Supplement: pgaf074_Supplementary_Data [file pgaf074_supplementary_data.zip › PNASNEXUS-PNASNEXUS-2024-01721-TR-s01.doc]

Electric field-coupled two-photon-polymerization system for on-demand modulation of 3D-printed structural color

Wei Feng^a^, Shurong Sheng^b^, Jiaqing He^a^, Xiaopu Wang^c^, Jiaqi Zhu^d^, Jiangfan Yu^c^, Jianhua Zhang^e^, Fan Wang^e^, Li Zhang^d,f^, Metin Sitti^e,g^

^a^CAS Key Laboratory of Mechanical Behavior and Design of Materials, Institute of Humanoid Robots, Department of Modern Mechanics, University of Science and Technology of China, Hefei 230027, Anhui, China

^b^Institute of Artificial Intelligence, Hefei Comprehensive National Science Center, Hefei 230088, China

^c^Shenzhen Institute of Artificial Intelligence and Robotics for Society (AIRS), The Chinese University of Hong Kong, Shenzhen 518129, Guangdong, China

^d^Department of Mechanical and Automation Engineering, The Chinese University of Hong Kong, Hong Kong, China

^e^Physical Intelligence Department, Max Planck Institute for Intelligent Systems, Stuttgart 70569, Germany

^f^Chow Yuk Ho Technology Center for Innovative Medicine, The Chinese University of Hong Kong, Hong Kong, China; Department of Surgery, The Chinese University of Hong Kong, Hong Kong, China; CUHK T Stone Robotics Institute, The Chinese University of Hong Kong, Hong Kong, China

^g^School of Medicine and College of Engineering, Koç University, Istanbul 34450, Turkey

**E-mail:** sitti@is.mpg.de, lizhang@mae.cuhk.edu.hk

**This PDF file includes:**

Figures S1 to S9

Table S1, S2

Movie S1

Figure S1. Electrically tunable structural color of heliconical cholesteric liquid crystals was investigated. Under high electric field strength, the reflected light shifted into the UV range, rendering it invisible. As the electric field decreased, the reflected light exhibited a red-shift, progressively transitioning to near-infrared wavelengths. When the electric field was insufficient to sustain the periodic assembly of heliconical cholesteric liquid crystals, the focal conic texture emerged. The relationship between electric field strength and structural color is illustrated in Figure 2D.

Table S1. Comparison of methods for multi-material 3D printing

| **Macroscale or microscale** | **Printing technique** | **Materials** | **Note** | **Literature** |
| --- | --- | --- | --- | --- |
| Macroscale | Digital light processing (DLP) | Colloidal photonic crystals |  | (1, 2) |
|  | Extrusion-based 3D printing | Thermoplastic materials， shear-thinning materials | Resolution is limited by the size of nozzles | (3-5) |
| **Microscale** | Step-by-step two-photon-polymerization (TPP) | Commercial resin, e.g., IP-S | Need careful realignment after every printing process | (6) |
|  | Microfluidics-integrated TPP | Acrylates |  | (7) |
|  | Magnetic fields-integrated TPP | Acrylates |  | (8, 9) |
|  | Electric fields-integrated TPP | Liquid crystals | Use electric fields to change the alignment of liquid crystals | (10, 11) |
|  | Electric fields-integrated TPP | Liquid crystals | Use electric fields to change the structural color of liquid crystals | **This work** |

**Table S2.** DFT-calculated dipole moment of LC mesogens.

| **Material** | **Chemical structure** | **Dipole moment** | **Method/basis set** |
| --- | --- | --- | --- |
| **5CB** |  | 4.92 Debye | B3LYP/  STO-3G |
| **S811** |  | 4.57 Debye | Hartree-Fock/  3-21G |
| **CB6A** |  | 5.04 Debye | B3LYP/  STO-3G |
| **CB7CB** |  | 4.49 Debye | B3LYP/  STO-3G |
| **RM257** |  | 0.35 Debye | B3LYP/  STO-3G |

DFT calculations were carried out with the Gaussian 09W (C.01) program.

Figure S2. Bubbling of the LC resin was induced by high writing power intensity.

Figure S3. No obvious phase separation was observed by using large scanning speed (12,000 µm/s).

Figure S4. Printed Taiji logo of different sizes. The dot in the size of 4 µm could be printed.

Figure S5. Electrical stability of structural color of the *polymerized* system. Due to the crosslinked network and anchoring force of the polymer network on liquid crystals, the structural color of polymerized LC polymer remained stable during the application of an electric field (2.6 V/µm, 1 kHz square wave), which was used to align unreacted monomers in the homeotropic alignment in the following step.

Figure S6. Design parameters in the comparison of writing speed using woodpile microstructure method and liquid crystal bottom-up method. (A) Parameters of the woodpile structure in the 50 µm-large cube. (B) 50 µm solid cube model for the liquid crystal bottom-up method.

For comparison, the potential of conventional helicoidal cholesteric liquid crystals to generate diverse structural colors by varying printing parameters was assessed. The monomers shown in Figure S8 were utilized in this evaluation. The results indicate that only a very narrow range of colors can be achieved with ordinary helicoidal cholesteric liquid crystals, highlighting the superior performance of the EF-TPP system in combination with heliconical cholesteric liquid crystals.

Figure S7. Chemical structures of conventional helicoidal cholesteric liquid crystal monomers used for the comparative investigation of phase separation. The mass ratio of monomers in LC mixtures is RM257:CB6A:RM105:LC756:5CB:IRG819=27:27.5:42.5:4:30:1.

Figure S8. Color evaluation of the ordinary helicoidal cholesteric liquid crystal system revealed a limited color range under varying scan speeds and laser power intensities (100% = 50 mW). Notably, the combination of high scan speeds and low laser power resulted in incomplete printing. In contrast, these findings underscore the advantages of combining heliconical liquid crystals with TPP for enhanced performance.

Figure S9. Crystallization behavior of polymerized samples was investigated. The cubic structures were fabricated via TPP, while the surrounding resin was polymerized using single-photon flood UV polymerization. Results revealed that crystallization occurred in the UV flood-exposed polymerized regions over time post-fabrication. In contrast, the TPP-printed microstructure regions remained non-crystalline, demonstrating their stability against crystallization.

**References**

1.	J. Liao *et al.*, 3D-printable colloidal photonic crystals. *Materials Today* **56**, 29-41 (2022).

2.	Y. Zhang *et al.*, Continuous resin refilling and hydrogen bond synergistically assisted 3D structural color printing. *Nature Communications* **13**, 7095 (2022).

3.	Z. Zhang, C. Wang, Q. Wang, Y. Zhao, L. Shang, Cholesteric cellulose liquid crystal ink for three-dimensional structural coloration. *Proceedings of the National Academy of Sciences* **119**, e2204113119 (2022).

4.	M. Doshi, A. Mahale, S. Kumar Singh, S. Deshmukh, Printing parameters and materials affecting mechanical properties of FDM-3D printed Parts: Perspective and prospects. *Materials Today: Proceedings* **50**, 2269-2275 (2022).

5.	N. M. Larson *et al.*, Rotational multimaterial printing of filaments with subvoxel control. *Nature* **613**, 682-688 (2023).

6.	Z. Liu *et al.*, Creating three-dimensional magnetic functional microdevices via molding-integrated direct laser writing. *Nature Communications* **13**, 2016 (2022).

7.	F. Mayer *et al.*, Multimaterial 3D laser microprinting using an integrated microfluidic system. *Science Advances* **5**, eaau9160 (2019).

8.	X. Hu *et al.*, Magnetic soft micromachines made of linked microactuator networks. *Science Advances* **7**, eabe8436 (2021).

9.	S. Zhang *et al.*, 3D-printed micrometer-scale wireless magnetic cilia with metachronal programmability. *Science Advances* **9**, eadf9462 (2023).

10.	A. Münchinger, L.-Y. Hsu, F. Fürniß, E. Blasco, M. Wegener, 3D optomechanical metamaterials. *Materials Today* **59**, 9-17 (2022).

11.	A. Münchinger *et al.*, Multi-Photon 4D Printing of Complex Liquid Crystalline Microstructures by In Situ Alignment Using Electric Fields. *Advanced Materials Technologies* **7**, 2100944 (2022).

**Legend for Movie S1**

Movie S1. Phase separation during two-photon-polymerization at a scanning speed of 5,000 µm/s with the laser power set as 40 mW.
